# To eat or not to eat: Reward delay impulsivity in children with loss of control eating, attention deficit / hyperactivity disorder, a double diagnosis, and healthy children

**DOI:** 10.1371/journal.pone.0221814

**Published:** 2019-09-16

**Authors:** Simone Munsch, Daniela Dremmel, Peter Wilhelm, Susanne Baierlé, Sophia Fischer, Anja Hilbert

**Affiliations:** 1 Department of Psychology, University of Fribourg, Fribourg, Switzerland; 2 Educational Department, Basel, Switzerland; 3 Integrated Research and Treatment Center Adiposity Diseases, Department of Medical Psychology and Medical Sociology, Department of Psychosomatic Medicine and Psychotherapy, University of Leipzig Medical Center, Leipzig, Germany; King's College London, UNITED KINGDOM

## Abstract

Reward delay impulsivity is a feature of attention deficit/hyperactivity disorder (ADHD) and a likely feature of loss of control eating (LOC-E), which might explain the higher risk of children with ADHD or LOC-E to become obese. The goal of this study was to investigate reward delay impulsivity in children with LOC-E, ADHD, or a double diagnosis, in contrast to healthy children. Children (8 to 13 years) with LOC-E (*n* = 24), ADHD (*n* = 33), a double diagnosis (*n* = 9), and healthy children (*n* = 34) performed a computer game (door opening task [DOT]) and the delay of gratification task (DoGT) to assess food related facets of reward delay impulsivity. In addition, children reported whether they worried to lose control over eating during the DoGT. There were no group differences in the DOT. However, children with ADHD or a double diagnosis had a significantly higher risk to eat prematurely during the DoGT than children with LOC-E, who were not significantly different from healthy children. Children with a double diagnosis were most likely to worry about losing control over eating during the DoGT, followed by children with LOC-E, and both had a significantly higher probability to worry than healthy children. For children with a double diagnosis the probability to worry was significantly higher than for children with ADHD. If replicated, these findings point to a special relevance of reward delay impulsivity in children with ADHD or a double diagnosis, compared to children with LOC-E. ADHD should be regularly assessed in children with LOC-E.

## Introduction

Loss of control eating (LOC-E) is defined as eating a large amount of food accompanied by a sense of loss of control over eating. LOC-E in children is associated with increased weight, shape, and eating concerns [1], more pronounced disinhibited or emotional eating, and an overall impaired mental health [2, 3]. LOC-E is associated with later onset of partial or full syndrome binge-eating disorder (BED) [3–5], overweight, and obesity [1]. According to a current meta-analysis almost every third child or adolescent with overweight or obesity suffers from LOC-E [6].

The core feature of LOC-E, the feeling of loss of control, is related to impulsivity [7, 8]. The impulsivity construct incorporates biological and psychological components. On a psychological level, impulsivity is characterized by insufficient planning or control of behavior [9, 10]. Two major subcomponents of impulsivity can be discriminated that are only weakly correlated [11]. The first, *rapid response impulsivity*, refers to inhibitory deficits that sacrifice accuracy of a behavior for speed. The second, *reward delay impulsivity*, refers to the preference of a smaller but immediate reward over a larger but delayed reward.

A facet of reward delay impulsivity is reward sensitivity [12, 13]. Gray [9] relates sensitivity to reward to an antagonistic model of motivation that consists of a behavior inhibition system (BIS) and a behavior activation system (BAS). While the BAS fosters the detection of rewarding stimuli [10], approach behavior and increases motor output, the BIS supports avoidance of punishment and decreases motor output. Previous literature shows that children with a higher reward sensitivity or delay impulsivity have an elevated risk to gain weight and to experience behavior, social, or school problems [14–17].

Reward delay impulsivity can be assessed with behavioral tests. The classic paradigm is the delay of gratification task (DoGT), in which children have to resist a small, immediately available reward (e.g. one marshmallow) in order to obtain a larger reward later (e.g. two marshmallows) [18]. Prior DoGT studies showed that children with obesity have more difficulties in delaying eatable rewards when compared to normal weight children [19–21]. However, when children were exposed to nonfood rewards, differences in DoGT were less clearly related to weight status [22, 23].

Meanwhile, computerized modifications of the DoGT paradigm based on token economics exist [11]. A computerized game, that assesses indicators of reward sensitivity, is the door opening task (DOT) [24, 25]. In the DOT paradigm, participants open virtual doors in order to gain points and finally earn a reward. However, with every additional door opened, the probability of earning points decreases. Therefore, those participants who continue opening doors despite decreasing chances to win are considered to be less sensitive for punishment and more sensitive for reward [10].

Two studies showed that overweight children (10−14 years) and obese, treatment seeking adolescents (12−15 years), who played the DOT, opened more doors than normal weight controls [26, 27]. Moreover, in the study of Nederkoorn et al. [26] adolescents with obesity, who had eating binges, opened more doors than those without binges. These results suggest, that children and adolescents with overweight or obesity and especially those with eating binges have an elevated reward sensitivity. However, Guerrieri and colleagues [10], who studied younger children (8−10 years) with the DOT, did not observe a difference between normal weight and overweight children. Whether this was due to the younger age of the children or to different reward conditions (apparently children did not receive an additional reward when they gained more points) remains open. Nevertheless, in this study, children with higher reward sensitivity ate more marshmallows in a bogus taste test that followed the DOT, especially when marshmallows varied in form, color, texture, and taste. Guerreri et al. [10] conclude that reward sensitivity might be a causal factor for overeating in an obesogenic environment. This reasoning might also apply to LOC-E, as suggested by the findings of Nederkoorn et al. [26]. Reward delay impulsivity is also crucial in attention deficit/ hyperactivity disorder (ADHD) that is characterized by age-inappropriate and excessive levels of inattention, overactivity, and impulsivity [28]. Studies that applied the classical DoGT paradigm confirmed the expectation that preschool children with ADHD symptoms have more difficulties to delay gratification than control children [29]. A current meta-analysis across 26 choice delay studies (mostly computerized DoGTs) clearly showed that children with ADHD stronger preferred immediate, smaller than delayed, larger rewards compared to healthy controls. This effect was larger for younger children (3−7 years; Hedges’ *g* = 0.83) than for older children (8−13 years; *g* = 0.46) and adolescents (*g* = 0.45) (14).

In sum, these findings show that reward delay impulsivity and reward sensitivity are psychopathological features of ADHD, and likely features of LOC-E. This might in part explain the higher risk of children with ADHD and LOC-E for overweight and obesity [30–34]. Despite its importance for the understanding of the pathological behavior in an obesogenic enviornment, reward delay impulsivity and reward sensitivity has not yet been investigated in children with LOC-E and compared to other clinical or healthy groups.

Consequently, we investigated reward delay impulsivity in children with LOC-E, ADHD, or both, and compared them to healthy children at the age of eight and 13 years, a developmental period when self-regulation consolidates [35]. On a behavioral level we assessed reward delay impulsivity using the DoGT [18] and reward sensitivity with the DOT [26] offering eatable and uneatable reward. Moreover, we captured the subjective experience of the children by assessing their worries to lose control while waiting during the DoGT.

The hypotheses (H) were as follows: Children with a mental disorder associated to increased impulsivity (LOC-E, ADHD, or a double diagnosis) have an elevated reward sensitivity and a reduced capability to delay gratification (H-1). They therefore open more doors in the DOT game (H-1a), have a higher probability to eat during the DoGT (H-1b), and more likely report worries to lose control over eating than healthy children (H-1c).

Although children with ADHD and LOC-E share the vulnerability of an impaired impulse control [36], food related loss of control is the core symptom of LOC-E. Children with LOC-E therefore have a higher sensitivity for food rewards and more difficulties to delay food rewards than children with ADHD (H-2). Consequently, compared to children with ADHD, children with LOC-E, open more doors in the DOT game, when a food reward is offered (H-2a) and have a higher probability to eat during the DoGT (H-2b). In addition, children with LOC-E report worries about losing control over eating more likely than children with ADHD (H-2c).

We further explored how children with ADHD and LOC-E differ from children with a single diagnosis. We expected that children with a double diagnosis have the most pronounced impulse-control deficits [37, 38] and the highest sensitivity towards reward and therefore, open more doors in the DOT than children with LOC-E, exploratory hypothesis (eH-3a), or ADHD (eH-4a). In addition, we expected that children with a double diagnosis have a higher probability to eat during the DoGT and report more worries about losing control over eating than children with LOC-E (eH-3b, eH-3c) or ADHD (eH-4b, eH-4c).

## Materials and methods

### Recruitment and sample

The current study was part of the Swiss University Study of Nutrition (SUN), investigating psychological and food intake related characteristics of children with LOC-E compared to ADHD and healthy controls [39–41]. Recruitment took place in 125 classes of 35 regular primary schools (3rd to 6th grade) in French and German speaking parts of Switzerland (Berne, Lausanne, Fribourg). Children and their parents were informed about the study aims to investigate eating behavior and development in families. Approval and written informed consent were obtained from the ethical committees of the cantons, the cantonal boards of education, the school boards, parents, and the children.

In an initial screening 1741 children and at least one of their parents completed questionnaires to assess symptoms of ADHD (Conners 3 AI) [42] and eating disorder psychopathology (ChEDE-Q) [43]. If children reported a LOC-E episode according to the ChEDE-Q or if parents reported that ADHD symptoms of their child were equal or above a Conners’ 3 AI T-score of 60, a telephone screening was conducted with children and parents in order to determine eligibility (*N* = 599). Altogether, 132 children and their parents were invited to attend a diagnostic session, where the Schedule for Affective Disorders and Schizophrenia for School-Age Children-Present and Lifetime Version (K-SADS-PL) [44] and the ChEDE [45] were applied.

Exclusion criteria for study participation were other eating disorders, compensatory behaviors (more than one episode during the past three months), ongoing treatment for overweight, medication with effects on eating behavior (except ADHD medication), serious medical conditions, as well as insufficient language skills. According to these criteria, 31 children were excluded from the study. One child stopped its participation during the experimental session.

Inclusion into the LOC-E group required at least three episodes of LOC-E during the last three months, accompanied by at least some degree of distress and two of five behavioral symptoms according to the ChEDE. Twenty-four children were included into the LOC-E group, of whom nine children fulfilled all criteria necessary for a BED diagnosis. Thirty-three children, who fulfilled the DSM-IV-TR [46] criteria for ADHD according to the K-SADS-PL were included into the ADHD group. Nine children simultaneously fulfilled all criteria for LOC-E and ADHD (with no child fulfilling criteria for a BED diagnosis). They were classified separately with a double diagnosis. Four children with ADHD and one child with a double diagnosis were prescribed with a stable ADHD medication which lasted at least three months, and was not changed for the study. The control group included 34 healthy children from the initial screening who were stratified to children in the LOC-E group for age, gender, and initially also for body mass index (BMI). Matching for BMI was later given up, because it became too difficult to find healthy children with overweight. Additional criteria for control children were the absence of lifetime or present LOC-E, of compensatory behaviors, of any symptoms of hyperactivity/ impulsivity and inattention, and of any mental disorder. Details about the characteristics of the final sample of 100 children are reported in Table 1.

**Table 1:** Sample characteristics and potential covariates.

	LOC-E (*n* = 24)		ADHD (*n* = 33)		LOC-E&ADHD (*n* = 9)		Healthy (*n* = 34)		Total (*N* = 100)	
	*M*	*SD*	*M*	*SD*	*M*	*SD*	*M*	*SD*	*M*	*SD*
Age	11.97	1.02	10.87	1.42	11.43	1.36	11.88	1.08	11.53	1.29
BMI	21.51	4.57	18.64	3.55	21.46	2.96	19.76	3.04	19.96	3.76
zBMI	0.78	1.26	0.21	1.15	1.09	0.62	0.44	0.92	0.51	1.09
Liking of food (1 not at all to 7 very much)	6.64	0.58	6.25	1.59	6.44	1.33	5.94	1.48	6.26	1.36
Sadness	1.61	1.31	1.28	0.73	1.44	1.01	1.30	0.73	1.38	0.92
Anxiousness	1.52	1.20	1.47	1.11	1.67	1.00	1.12	0.33	1.38	0.94
BMI > 90th percentile	10	41.7	7	21.2	3	33.3	5	14.7	25	25
Gender (Girls)	16	66.7	14	42.4	4	44.4	23	67.6	57	57
Language (German)	10	41.7	24	72.7	4	44.4	22	64.7	60	60
Order of reward condition in DOT-game (eatable first)	9	37.5	16	48.5	8	88.9	18	52.9	51	51

LOC-E = loss of control eating; ADHD = attention deficit / hyperactivity disorder; LOC-E&ADHD = double diagnosis; BMI = body mass index, zBMI = standardized body mass index.

### Procedure and measures

Parents (89 mothers, 10 fathers, and 1 time both parents) and their children arrived in the laboratory at 3 pm, after having eaten lunch at home. After the diagnostic interview, children chose their preferred snack food, performed the DOT and then the DoGT. Finally, body weight and height were measured.

Assessment of psychological problems and mental disorders. LOC-E was assessed by the *Eating Disorder Examination adapted for Children* (ChEDE [45]; German version [43]; French version translated by the authors). The ChEDE has a satisfying internal consistency and interrater reliability [47].

*The Conners 3 ADHD Index (Conners 3AI* [42]; German version [48]; French version translated by the authors) for parents was used to identify children with ADHD in the initial screening. It is a reliabile and frequently used screening instrument that has demonstrated high discriminant validity in different populations.

*The Schedule of Affective Disorders and Schizophrenia for School age children–Present and Lifetime Version* (K-SADS-PL; German version [44]; French version [49]) was applied to assess mental disorders of children and to evaluate whether children with symptoms of ADHD fulfilled diagnostic criteria. The K-SADS-PL is a reliable structured interview for diagnoses according to DSM-IV-TR that has demonstrated its validity in different populations, and was repeatedly used to identify ADHD in children and adolescents [50]. In the revision of the DSM, diagnostic criteria for ADHD were only slightly modified. The most relevant change was that onset of symptoms can be later in DSM-5 (before age 12) than in DSM-IV-TR (before age 7). Therefore, our ADHD classification is stricter regarding age of onset, but still compatible with DSM-5 criteria [28].

*Interviewers* were master and PhD students in Psychology who were trained at the Department of Psychiatry, University of Lausanne for the K-SADS-PL and by the first and last author for the ChEDE. All interviews were coded twice. Disagreements were resolved in supervision meetings with the first and last authors.

#### Computerized door opening task (DOT)

The DOT version used in the current study was from Nederkoorn et al. [26]. The DOT is a game of chance in which the initially high probability to win points decreases continuously. Participating children decide whether they open a virtual door which is presented on the computer screen, by clicking a “yes” or “no” button. If a child opened the door and a smiling face appeared she or he would gain one point. However, if a sad face appeared she or he would lose a point. The game was over when the child refused to open another door, or when the maximum of 90 doors had been opened. The game started with a credit of 10 points. The probability to win an additional point by opening a door was 90 percent for the initial 10 doors, it decreased to 80 percent for the next 10 doors, then to 70 percent and so forth until 10 percent was reached for the last 10 doors. The probability to lose a point was reciprocal; it increased from 10 to 90 percent. After 30 doors were opened, winning doors appeared before losing doors for every block of 10 doors. After 45 doors were opened, the maximum number of 35 points was attained.

Children played the DOT twice: once with food rewards (chocolate, sweets, apples, or mandarins) and once with nonfood rewards (colored pencils, felt-, or light pens). The order of the reward conditions was counterbalanced. After children had seen the reward items, the DOT was explained and children could familiarize with the procedure by opening several test doors. They were instructed to gain as many points as possible and that they would earn more rewards with more points. At the end of each run, children received three reward items when they gained 25 or more points and two items when they gained less. The measure for reward sensitivity was the number of doors opened. In addition, we examined the points achieved in each condition.

#### Delay of gratification task (DoGT)

Prior to the DoGT, the experimenter invited the child to self-report current affective state on a visual analogue Scale (VAS) including the two items *“do you feel sad*?*”* and *“do you feel anxious*?*”* (1 = “not at all” to 7 = “very much/extremely”).

First, the experimenter presented three plates with small samples (about 7g) of chocolate cookies, chips, and gummy candies. Children could smell and taste each snack and see the original packages (175−200g). Then they were asked how much they like each snack (1 = “not at all” to 7 = “very much”) and how many packages (1, 2 or 3) of the most preferred snack they would like to get.

Later (after the DOT) children were exposed to a slightly modified version of the DoGT [18]. A plate with the child’s preferred snack, in addition to the original package with a rest (1/8) of the snack was put in front of the child. The experimenter said that she would leave for an urgent telephone call and would later return with packages of the preferred snack. She said, that the child could eat from the snack immediately. However, the child would only receive the desired quantity of the preferred snack, if he or she could resist eating until the experimenter’s return, which was after 15 minutes. Children who began to eat were allowed to eat the rest of the snack. Children who could resist eating received the desired quantity of the preferred food. The entire sequence was video-taped. The measure of delay of gratification was the video based coding whether a child ate or not before the experimenter returned. Two children, who picked cookie crumbs from the plate, but did not eat from the cookie itself were rated as having eaten.

Immediately after the DoGT, children answered the following question: *“Did you worry to lose control over eating while waiting*?*”* (1 = “not at all” to 7 = “very much”). We dichotomized the variable because it was heavily skewed. Due to an administrative error, six children did not complete these questions after the DoGT, and sample size for this variable and for liking-ratings of snacks chosen was reduced to 94.

#### Standardized body mass index (zBMI) and classification of normal weight vs. overweight

Children’s weight and height were measured using a standardized balance and a stadiometer. Using German norm data [51] each child’s BMI (weight in kg /height in meter^2^) was transferred into the corresponding age (in months) and gender related z-value (zBMI). Based on these *z*-values percentiles were computed. Children with a zBMI > 1.28, which corresponds to a BMI higher than 90 percent of their peers (pBMI > 90%), were classified as being overweight.

### Statistical analysis

We used one way ANOVAs to identify potential control variables and to determine differences between diagnostic groups in children’s age, zBMI, and their liking of food chosen for the DoGT. We explored whether groups differed by gender, weight status, and the order in which reward conditions were administrated in the DOT game, using Fisher’s exact tests. To obtain a stricter test for the assumption that there were no differences between groups of at least medium size (*f* = 0.25; *w* = .30) we relaxed α to .2 (two tailed). The resulting power (computed with GPower 3.1.9.2) was .87 for the χ^2^ test (approximation for Fisher’s exact test for 4 groups) and .68 for the ANOVA.

To test the hypotheses related to the DOT, we computed a two-factorial analysis of covariance for repeated measures (2 × 4). Dependent variables were the number of doors opened in each reward condition. Type of reward condition (food vs. nonfood) was the within subject factor, and the diagnostic groups were the between subject factor. We controlled all variables that could vary between groups: age (centered around the mean of 11.5 years), gender (male = 0, female = 1), weight status (dummy coded; overweight or obesity [pBMI > 90%] = 1), and the order of reward conditions (nonfood first, food second = 0; food first, nonfood second = 1). We estimated interaction effects between all covariates and the within subject factor. We calculated the same analysis to explore whether the points achieved during the DOT were different between groups or conditions. Power was sufficient (.80, α = .05, Rho = .50) to detect moderate to large between-subject effects (*f* = .30), and small to moderate within-subject (*f* = .14) and interaction effects (*f =* .21).

We calculated logistic regression models (LR) to predict whether children ate during the DoGT and whether children reported worries to lose control while waiting. Both criterion variables were regressed on the same set of predictors. First, we entered control variables that differed between groups: age, gender, and weight status (model 1). In a second step, we tested whether children with a mental disorder differed from healthy children (H-1b; H-1c) by entering a dummy variable that was coded 0 for patients and 1 for healthy controls (model 2). In a third step, we added a dummy variable for children with ADHD and another for children with a double diagnosis (model 3.0). In this model, children with LOC-E were the reference group. Therefore, the coefficients indicate how healthy children, children with ADHD, and children with a double diagnosis differ from children with LOC-E, and thus provide a specific test for the corresponding hypotheses (H-2b, H-2c, eH-3b; eH-3c). To test whether a particular diagnostic group differs from healthy children, we recalculated model 3.0, using the dummy variables of the three diagnostic groups (LOC-E, ADHD, double diagnosis) as predictors and healthy children as the reference group (model 3.1). In addition, we explored whether children with a double diagnosis have a higher probability to eat or to worry than children with ADHD (eH-4b, eH-4c), using children with ADHD as the reference group in model 3.2. Models 3.0, 3.1, and 3.2 are essentially equal, except that the coefficients of the groups contain different information, due to the particular reference group.

We report Nagelkerke’s *R*^*2*^ (*R*^*2*^_*N*_) for each model. Because *R*^*2*^_*N*_ provides only an approximation of the variance explained it cannot be used to calculate *R*^*2*^_*change*_. Tabachnik and Fidell [52] suggest calculating *R*^*2*^ directly from the data by regressing the criterion variable on the predicted values of a given model (p. 460). We followed this advice and report *R*^*2*^_*change*_ based on Tabatchnick and Fidell’s *R*^*2*^ as an estimate of the variance that is explained in the criterion variable by the set of predictors added to the model at a particular step of the analysis. The corresponding χ^2^ test indicates whether the additional variance explained is beyond chance. As an approximation of the sensitivity of our analyses to discover a certain increase in variance we used the *R*^*2*^-increase module for multiple regression analysis of Gpower. Power was sufficient (.80, α = .05) to detect a moderate increase in unexplained variance (*f* ^2^ = .08, when one predictor was added, and *f* ^2^ = .10, when a set of two predictors was added). To test whether a single coefficient was different from zero we used the corresponding Wald χ^2^ test with one *df* (α = .05).

## Results

### Differences between diagnostic groups and associations between variables

All children liked the food they chose for the DoGT equally well, *F*(3, 90) = 1.22, *p* = .309, η^2^ = .039. However, there were significant differences between groups in age, *F*(3, 96) = 5.27, *p* = .002, η^2^ = .141; and potential differences (*p* < .2) in the gender distribution, Fisher's Exact Test = 5.85, *p* = .117, *w* = 0.24; zBMI, *F*(3, 96) = 2.27, *p* = .086, η^2^ = .066; percentage of overweight children, Fisher's Exact Test = 5.96, *p* = .104, *w* = .24; and order of reward condition in the DOT game, Fisher's Exact Test = 7.04, *p* = .069, *w* = .27 (see Table 1). Consequently, weight status, age and gender were controlled in all analyses. The last result indicates that randomization of the order of reward conditions in the DOT game did not lead to equal proportions across diagnostic groups. It was therefore used as the fourth covariate in the analysis of DOT measures.

The number of doors opened in the two DOT-game conditions were positively correlated with each other (*r* = .38, *p* < .001) and negatively correlated with the points achieved in the same reward condition (nonfood rewards: *r* = -.45, *p* < .001; food rewards: *r* = -.39, *p* < .001). Thus children who opened less doors earned more points. In addition, points achieved in the food reward condition negatively correlated with the probability to eat during the DoGT (*r* = -.32, *p* = .001). Thus, children who gained fewer points during the eatable reward condition of the DOT game had a higher probability to eat during the DoGT. However, there were no other significant associations between dependent variables of our study (probability to eat during the DoGT, probability to report worries to lose control over eating, doors opened or points achieved in the two DOT conditions: *rs* ≥ -.05, *ps* ≥ .605 and *rs* ≤ .16, *ps* ≥ .110).

### Number of doors opened and points achieved during the DOT

Regarding covariates, there was a moderate and significant main effect of the order of reward conditions on the number of doors opened in the DOT game, *F* (1, 92) = 4.97, *p* = .028, η_p_^2^ = .051. Parameter estimates show that those children who got food rewards first and nonfood rewards second, opened approximately 10 doors less in both conditions compared to children who got nonfood rewards first and food rewards second. There were no significant main or interaction effects of age, gender, or weight status, *Fs* (1, 92) ≤ 3.16, *ps* ≥ .079, ηs_p_^2^ ≤ .033. Furthermore, there was no significant difference between reward conditions, *F* (1, 92) = 1.27, *p* = .263, η_p_^2^ = .014, nor a significant effect of diagnostic groups, *F* (3, 92) = 0.09, *p* = .964, η_p_^2^ = .003, or a significant interaction between diagnostic groups and reward conditions *F* (3, 92) = 2.15, *p* = .099, η_p_^2^ = .066. The latter results and the pattern of means, shown in Table 2, suggest the rejection of all hypotheses (H-1a, H-2a, eH-3a, and eH-4a).

**Table 2:** Descriptive results of the door opening task (DOT).

	ADHD (*n* = 33)		LOC-E (*n* = 24)		LOC-E&ADHD (*n* = 9)		Healthy (*n* = 34)		Total (*N* = 100)	
	*M*	*SD*	*M*	*SD*	*M*	*SD*	*M*	*SD*	*M*	*SD*
Number of doors opened										
- nonfood rewards	55.12	28.51	55.17	26.53	39.78	15.57	41.76	30.08	49.21	28.19
- food rewards	47.06	23.85	42.75	22.93	50.56	29.31	46.41	27.60	46.12	25.18
- average across both reward conditions	51.09	21.17	48.96	21.31	45.17	18.61	44.09	24.77	47.67	22.18
Points achieved										
- nonfood rewards	22.15	8.35	23.58	9.44	29.33	4.36	21.76	8.09	23.01	8.43
- food rewards	25.79	7.96	26.92	8.19	23.22	8.48	24.32	9.17	25.33	8.45
- average across both reward conditions	23.97	5.94	25.25	6.90	26.28	4.93	23.04	6.60	24.17	6.33

LOC-E = loss of control eating; ADHD = attention deficit / hyperactivity disorder; LOC-E&ADHD = double diagnosis; BMI = body mass index, zBMI = standardized body mass index.

We examined the points gained by children. Again, there was no significant difference between reward conditions, *F* (1, 92) = 2.65, *p* = .107, η_p_^2^ = .028, but the interaction between reward conditions and the order of their presentation was significant, *F* (1, 92) = 4.15, *p* = .044, η_p_^2^ = .043. Parameter estimates showed that when nonfood rewards were offered first, children gained almost six points more in the second run when food rewards were offered, but when food rewards were offered first, there was no difference between the two conditions. However, there were no significant differences between diagnostic groups, *F* (3, 92) = 1.25, *p* = .296, η_p_^2^ = .039, nor was there a significant interaction of diagnostic groups and reward conditions, *F* (3, 92) = 1.20, *p* = .314, η_p_^2^ = .038.

### Children’s probability to eat during the DoGT

Children’s gender, age, and weight status, entered as control variables in the first step, did not significantly explain variance in the probability to eat during the DoGT, model 1: *R*^*2*^_*N*_ = .080, *R*^*2*^_*change*_ = .048, χ^2^ (3, *N* = 100) = 5.59, *p* = .133. Contrary to the prediction of H-1b, the distinction between healthy children and children with LOC-E, ADHD or both did not help to explain additional variation in the probability to eat, model 2: *R*^*2*^_*N*_ = .082, *R*^*2*^_*change*_ = .003, χ^2^ (1, *N* = 100) = 0.19, *p* = .661. However, adding the dummy coded variables for ADHD, and double diagnosis in a next step led to a significant increase in the variance explained, model 3.0: *R*^*2*^_*N*_ = .195, *R*^*2*^_*change*_ = .088, χ^2^ (2, *N* = 100) = 8.47, *p* = .014.

**Fig 1:**
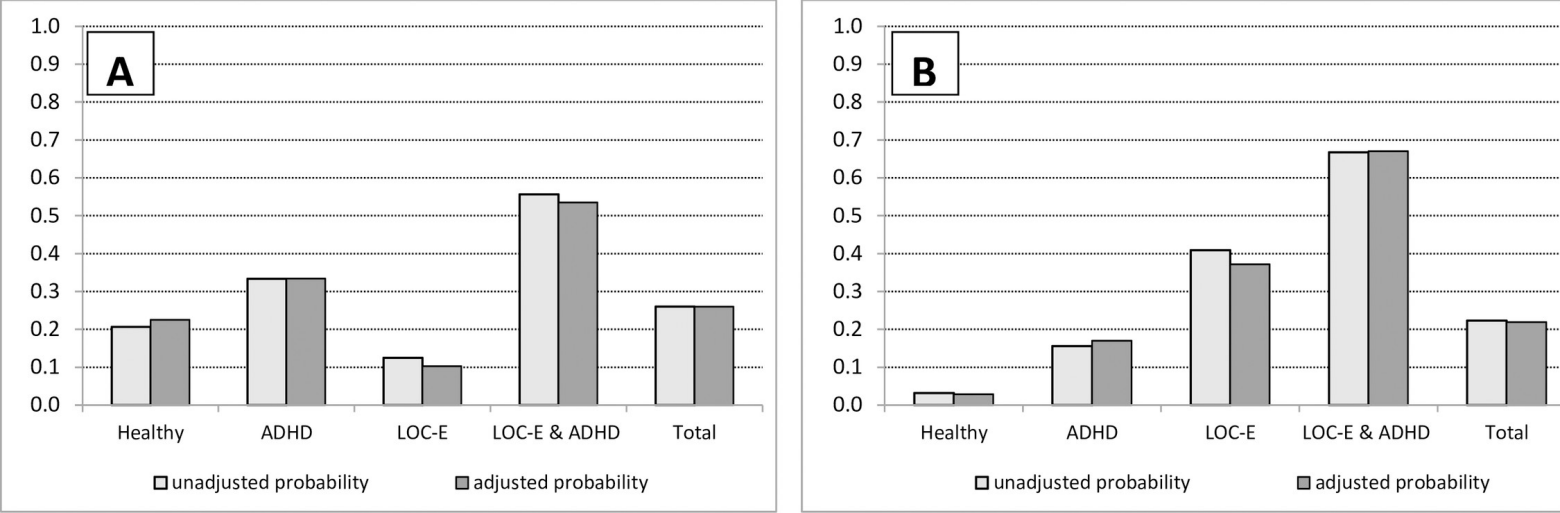
**Unadjusted and adjusted probabilities (A) to eat during the delay of gratification task (DoGT), (B) to worry to lose control over eating.** (A) Unadjusted probabilities to eat are based on the percentage of children with premature eating in each group during the DoGT (divided by 100). The adjusted probabilities were estimated from the coefficients presented in Table 3. (B) Unadjusted probabilities to worry are based on the percentage of children who did not deny the question *“*did you worry to lose control over eating while waiting?*”* (divided by 100). The adjusted probabilities were estimated from the coefficients presented in Table 4. Estimates for the adjusted probabilities were computed separately for male and female children with and without overweight, and averaged across gender. Finally, the weighted average for children with and without overweight was computed according to the proportion in the sample. 11.5 years was the average age of the children and the value on which the control variable age was centered. Therefore, the adjusted probabilities refer to 11.5 years old children. ADHD = attention deficit / hyperactivity disorder; LOC-E = loss of control eating; LOC-E & ADHD = double diagnosis.

Although covariates did not explain a significant amount of variance when they were entered together, the coefficients of the final model (see Table 3) show that weight status had a significant influence. Children who had overweight or were obese had a higher probability to eat than children with normal weight. However, for none of the diagnostic groups the probability to eat was significantly different from that of healthy children, which is again incompatible with H-1b. In addition, contrary to H-2b, the likelihood to eat was higher for children with ADHD than for children with LOC-E (model 3.0, LOC-E was reference: *B* = 1.63, *SE* = 0.81, Wald-χ^2^ *=* 4.10, *p* = .043). Raising *e* to the power of the coefficient (Exp(*B*)) reveals the odds ratio (*OR*). The *OR* was 5.10. It indicates that the probability to eat relative to the probability to resist eating was about five times higher for children with ADHD than for children with LOC-E. However, the 95% confidence interval (*CI*) of the *OR* was between 1.05 and 24.70, informing us that the uncertainty of the *OR* estimate was large. Although children with a double diagnosis were not significantly different from children with ADHD (model 3.2, ADHD was reference: *B* = 0.93, *SE* = 0.81, Wald-χ^2^ *=* 1.31, *p* = .252, *OR* = 2.53, 95% *CI*: 0.52, 12.43), they had a significantly higher probability to eat than children with LOC-E (model 3.0, LOC-E was reference: *B* = 2.56, *SE* = 0.98, Wald-χ^2^ *=* 6.81, *p* = .009, *OR* = 12.93, 95% *CI*: 1.89, 88.36). While the latter result is in line with the corresponding exploratory hypothesis (eH-3b), the former is not (rejection of eH-4b). For a graphical display of the unadjusted and adjusted probabilities to eat see Fig 1A.

**Table 3:** Predicted probability to eat during the DoGT. Coefficients of the logistic regression analysis with healthy children as the reference group (Model 3.1).

	*B*	*SE*	*Wald-χ*^*2*^	*p*-Value	Exp*(B)*	95% *CI*
	-2.13	0.64	11.01	.001	0.12	(-3.40, -0.86)
Covariates						
- Age (centered at 11.5 years)	-0.18	0.21	0.75	.387	0.83	(0.55, 1.26)
- Gender (Female)	0.81	0.57	2.03	.154	2.25	(0.74, 6.89)
- BMI > 90th percentile	1.39	0.59	5.58	.018	4.02	(1.27, 12.78)
Diagnostic groups						
- LOC-E	-1.01	0.80	1.60	.206	0.36	(0.08, 1.75)
- ADHD	0.62	0.63	0.96	.328	1.85	(0.54, 6.35)
- Double diagnosis	1.55	0.84	3.37	.066	4.69	(0.90, 24.40)

LOC-E = loss of control eating; ADHD = attention deficit / hyperactivity disorder; BMI = body mass index; Exp(*B*) *= e* raised to the power of *B*, equals odds ratio*; CI =* Confidence Interval.

### Children’s worry to lose control over eating

Children’s gender, age, and weight status, entered in the first step, did not explain a significant proportion of variance in children’s likelihood to worry to lose control, model 1: *R*^*2*^_*N*_ = .032, *R*^*2*^_*change*_ = .022, χ^2^ (3, N = 94) = 1.97, *p* = .579. As predicted by H-1c, there was a significant difference between healthy children and children with LOC-E, ADHD, or both, model 2: *R*^*2*^_*N*_ = .224, *R*^*2*^_*change*_ = .120, χ^2^ (1, *N* = 94) = 12.95, *p* < .001. In addition, adding the dummy coded variables for ADHD, and double diagnosis significantly increased the variance explained, model 3.0: *R*^*2*^_*N*_ = .331, *R*^*2*^_*change*_ = .113, χ^2^ (2, *N* = 94) = 8.02, *p* = .018. This indicates that there were substantial differences among the diagnostic groups.

Coefficients and *ORs* of model 3.1, in Table 4, show that compared to healthy children, the probability to worry relative to the probability not to worry was not significantly higher for children with ADHD, but twenty times higher for children with LOC-E, and even 70 times higher for children with a double diagnosis. The latter results were in line with the prediction of H-1c. Direct comparisons among diagnostic groups revealed that children with LOC-E were not significantly different from children with ADHD (model 3.2, ADHD was reference: *B* = 1.08, *SE* = 0.71, Wald-χ^2^ *=* 2.28, *p* = .131, *OR* = 2.94, 95% *CI*: 0.73, 11.90), which does not correspond to H-2c. Children with a double diagnosis had a significantly higher probability to worry to lose control over eating than children with ADHD, (model 3.2, ADHD was reference: *B* = 2.33, *SE* = 0.88, Wald-χ^2^ *=* 7.07, *p* = .008, *OR* = 10.32, 95% *CI*: 1.85, 57.61), but they were not significantly different from children with LOC-E (model 3.0, LOC-E was reference: *B* = 1.26, *SE* = 0.87, Wald-χ^2^ *=* 2.08, *p* = .149, *OR* = 3.51, 95% *CI*: 0.64, 19.35). While the former result was predicted by eH-4c, the latter result did not correspond to eH-3c. None of the control variables had a significant effect. For a graphical display of the probabilities to worry see Fig 1B.

**Table 4:** Predicted probability of worries to eat during the DoGT. Coefficients of the logistic regression analysis with healthy children as the reference group (Model 3.1).

	*B*	*SE*	*Wald-χ*^*2*^	*p*-Value	Exp*(B)*	95% *CI*
Constant	-3.84	1.16	10.96	.001	0.02	(-6.14, -1.54)
Covariates						
- Age (centered at 11.5 years)	0.48	0.65	0.54	.462	1.62	(0.45, 5.81)
- Gender (Female)	0.08	0.25	0.11	.738	1.09	(0.67, 1.76)
- BMI > 90th percentile	0.26	0.67	0.15	.695	1.30	(0.35, 4.82)
Diagnostic groups						
- LOC-E	3.00	1.12	7.19	.007	20.03	(2.24, 179.28)
- ADHD	1.92	1.16	2.74	.098	6.82	(0.70, 66.17)
- Double diagnosis	4.25	1.27	11.17	.001	70.32	(5.81, 851.58)

LOC-E = loss of control eating; ADHD = attention deficit / hyperactivity disorder; BMI = body mass index; Exp(*B*) *= e* raised to the power of *B*, equals odds ratio*; CI =* Confidence Interval.

## Discussion

ADHD and BED in childhood and adolescence, but also LOC-E as a frequent precursor of BED are characterized by an elevated impulsivity [11]. An elevated impulsivity in an environment where palatable food is always available, facilitates weight gain, and contributes to the higher risk for children with ADHD, BED, or LOC-E to become overweight or obese [10, 30, 31, 34].

Although impulsivity seems to be a crucial feature of ADHD and LOC-E, studies that have directly compared facets of impulsivity between these groups are rare, and in none of these studies reward sensitivity or reward delay impulsivity have been investigated with food specific incentives. Therefore, the goal of the current study was to investigate whether children with LOC-E differ from children with ADHD in their level of reward sensitivity, reward delay impulsivity, and their probability to worry to lose control over eating (H-2a, b, c), and whether these two groups differ from healthy children, especially when palatable food specific incentives are offered (H-1 a, b, c). In order to ensure an unambiguous interpretation of results, we did not allow symptoms of LOC-E or ADHD to overlap. Instead we treated those children who fulfilled criteria for LOC-E and ADHD at the same time as a separate group. This gave us the opportunity to explore, whether children with a double diagnosis, who are supposed to have more severe problems, differ from children with a single diagnosis. Moreover, in addition to age and gender, we controlled for weight status (normal vs. overweight or obese) in our analyses. This allows us to attribute results to the children’s psychopathological features of their group, and not to their weight status, which is associated with reward sensitivity [26, 27] and reward delay impulsivity [17, 19, 21].

### Lacking groups differences in the DOT

Contrary to our hypotheses that the level of reward sensitivity increases from healthy children to children with ADHD, and children with LOC-E to children with a double diagnosis (H-1a, H-2a, eH-3a, eH-4a), we did not find a significant difference between the four groups neither in the number of doors opened nor in the number of points achieved. Moreover, we did not find a main effect of reward conditions (nonfood vs. food rewards), nor a significant interaction of reward conditions with groups. The only significant effect of modest size was between children who got food rewards first and those who got them second. This shows that our attempt to produce equal groups for the order of reward conditions failed for children with LOC-E and a double diagnosis because these two groups were not separately considered for randomization of the order of reward conditions (see Table 1). Although this impairs conclusions about the effects of food vs. nonfood rewards, it does not affect the interpretation of the main effect groups.

Our results are in contrast to those of other researchers who found that compared to healthy controls, children or adolescents with eating binges and obesity, or ADHD opened more doors when playing the DOT [24–27, 53]. We see two reasons that might explain why we did not observe differences between groups.

The first reason is related to the rewards offered. In each condition children were promised to receive more rewards if they gained more points. At the end of each trial, children then got three items if they gained 25 or more points and two items if they gained less. This plan of rewards might not have motivated children to gain as many points as possible. Motivation would be better fostered if the relationship between rewards offered and points achieved was strengthened (e.g. one reward for every point) and this clearly communicated before the task. A closer relationship between points achieved and rewards offered might then better reveal potential differences in reward sensitivity between groups.

The second reason is related to the construct validity of the DOT. In order to have a high score it is necessary to infer the rules underlying the DOT. This is not simple, because the rules are not obvious. In every block of 10 presented doors children first win and then lose points, while the number of losing doors increases from block to block. Children need to recognize that the relationship between winning and losing doors changes every 10 doors. Despite that, children could assume that there might be a turning point when more points could again be gained. Therefore, continuing to open doors until the end of the game is a reasonable strategy, at least once. In fact, our data show that 15 children, who opened every door in the first trial, did not open every door in the second trail. However, eight children who did not open every door in the first trial did so in the second. Only six children opened all doors in both trials. These findings support our reasoning that children try to explore the game to figure out the underlying rules.

On average, children opened two doors less in the second trial. Although this change was not statistically significant, it led to a significantly higher number of points won in the second trial. Therefore, we conclude that the number of doors opened in the DOT might reflect reward sensitivity, but also depends on logical problem solving skills, and familiarity with the game. Future studies should include the assessment of global cognitive functioning of children in order to approximate the effects of e.g. inductive reasoning on findings in the DOT and to determine whether the number of doors opened in the DOT or the score achieved, are indeed valid measures of response perseveration and reward sensitivity.

### Group differences in the DoGT

Children’s behavior during the DoGT was in part in line with our expectations and previous research. We controlled for weight status and found that the probability to eat relative to the probability to resist eating was four times higher for children with overweight or obesity, compared to children with normal weight (see Table 3). Other researchers reported similar findings [19–23].

As expected (eH-3b, H-1b), children with a double diagnosis had a significantly higher probability to eat prematurely than children with LOC-E and healthy children (the *p-*value for the comparison with healthy children was .066 and could be interpreted as a trend; see Fig 1A). *OR*s of 12.9 and 4.6 indicate that these effects were rather large. Children with a double diagnosis also had a somewhat higher probability to eat than children with ADHD (OR = 2.5), which was however not significant (no support for eH-4b). Together these results suggest, in line with Meule [37] and Nazar et al. [38], that children with a double diagnosis have the most difficulties to delay rewards, and seem to have a substantially higher reward delay impulsivity than children with LOC-E.

Although children with ADHD did not have a significantly higher probability to eat than healthy children, the observed difference was in the expected direction (H-1b). The size of the effect (OR = 1.9) was small to moderate but comparable to the effect reported in preschool children with symptoms of ADHD by von Stauffenberg and Campbell [29]. They obtained a significant result because their sample was several times larger than ours, and consequently powerful enough to detect even rather small effects.

In their meta-analysis, Patros et al. [11] found a big effect when small children with ADHD were compared to healthy children and a medium effect when older children–like in our study–were compared. The bigger effect sizes, compared to our results and those of von Stauffenberg and Campbell, might be due to the fact that Patros et al. included only computerized DoGT studies. These might produce larger effects than studies based on the classic DoGT paradigm.

Interestingly and contrary to our expectations, children with LOC-E had the lowest probability to eat, and were not significantly different from healthy children (rejection of H-1b). Moreover, they had a significantly lower probability to eat than children with ADHD (the OR indicated a moderate to large effect and suggests a clear rejection of H-2b) and children with a double diagnosis (as discussed before the latter effect was large and in line with expectation eH-3b). This pattern of results suggests that the driving force to eat prematurely in the DoGT situation is the presence of ADHD symptoms. LOC-E only seems to matter when it occurs together with ADHD, but then it increases reward delay impulsivity.

We think that there are two plausible reasons, why children with LOC-E did not eat prematurely during the DoGT. Firstly, children with LOC-E might have been more affected by the experimental situation than children in the other groups. Because LOC-E or binge eating attacks trigger feelings of shame and guilt, persons try to conceal their symptomatic behavior. Therefore, LOC-E or binge eating episodes usually occur when persons are alone and feel unobserved [28, 54]. In our experiment, children were informed about the study content to investigate eating behavior and development and saw the video camera in the room and knew that they were observed. In addition, they knew that the experimenter would come back and check whether they had eaten. Under these circumstances they could exhibit a higher control over eating than children with ADHD or a double diagnosis, who suffer from an impaired inhibitory behavior control. Consequently, children with LOC-E’s capability to resist eating during the DoGT is probably not a valid indicator for their capability to control eating when they are being unobserved at home. Future studies should include ambulatory assessment techniques in daily life to investigate children’s natural behavior [55]. Secondly, our study design favored the induction of reward delay impulsivity but did probably not induce eating to reduce aversive affective states, named emotional eating. Such emotional eating is known to be an important trigger of LOC [41]. Futures studies could specify the interaction of reward delay impulsivity and emotional eating in LOC-E and ADHD compared to healthy controls.

### Group differences in the worries to lose control while waiting

In contrast to our computer based and behavioral measures of reward sensitivity and reward delay impulsivity, the pattern of results regarding children’s *self-reported worries to lose control over eating while waiting* was as expected (see Fig 1B). Differences between groups were at least of moderate size (ORs > 2.9) and all in the expected direction. However, due to the limited sample size only rather large differences (ORs > 10) became significant. Children with any diagnosis worried significantly more than healthy children (when we interpret *p* = .098 of children with ADHD as a trend), and children with a double diagnosis worried significantly more than children with ADHD (in line with H-1c and eH-4c).

The experience of loss of control when eating is the crucial symptom of LOC-E. Our results show that a tempting situation like the one we realized in our study more likely triggers worries about such a loss of control in children with LOC-E and a double diagnosis. It has to be further investigated, whether such worries might trigger emotional eating to cope with aversive affective states and how this interaction might lead to binge eating [56, 57].

In our study, self-reported worries to lose control and behavioral measures were independent of each other. Despite one exception, there were also no significant correlations between DoGT and DOT measures. Weak associations between measures of similar or closely related constructs − like reward sensitivity and reward delay impulsivity − are puzzling but quite common [26, 27, 58].

### Limitations

The major limitation of our study was the small sample size of groups, especially the small number of children with a double diagnosis. As a consequence, estimates had large confidence intervals. Even though we partly found large, significant effects between groups, it has to be considered that we did not adjust alpha levels for multiple testing.

Second, it has to be kept in mind that we included a French and German bilingual study group, which is why we had to translate questionnaires within our bilingual team, but did not validate them before first usage out of feasibility reasons. Thus, we were not able to assess validity and reliability of the French questionnaires and the interview. The diagnostic procedure in this study was based on questionnaire screening, followed by standardized interviews with parents and children. However, we did not get information from teachers, which would have strengthened the validity of the ADHD diagnosis. Third, children on medication concerning symptoms of ADHD were not excluded in our study. Future studies might consider including them only after a wash-out period prior to the laboratory tasks. Forth, we recruited children with LOC-E, where symptomatology might not in all children be fully developed. However, LOC-E is of importance, because subthreshold eating disorder symptoms are associated with levels of impairment and distress that are comparable to those of a fully developed BED [59]. Moreover, the likelihood is high that children with LOC-E develop a BED or become overweight or obese [1, 3–5, 59]. Finally, like in every quasi-experimental study our findings are correlative and do not allow causal conclusions. Therefore, longitudinal studies should investigate causal pathways: For example, whether increased worries to lose control, when faced with the delay of food intake, predict future frequencies of binge eating episodes [60].

## Conclusion

Our study is the first that compared behavioral measures of reward delay impulsivity, reward sensitivity and subjectively experienced worries to lose control between children with LOC-E, ADHD, or a double diagnosis, and healthy children. From our findings, we assume that the DOT does not seem to provide valid measures of reward sensitivity and this requires further investigation. We further suggest from results based on the DoGT that the lack of behavioral inhibition associated with ADHD symptoms is the driving force to eat prematurely. In contrast, symptoms of LOC-E do not seem to facilitate premature eating. The fact that children knew that they were observed was probably the cue that allowed especially children with LOC-E to exhibit a high level of control. However, those children who had LOC-E and ADHD symptoms at the same time had the highest probability to eat prematurely, probably because the strong desire to eat associated with LOC-E could not be adequately controlled due to the decreased capability to inhibit behavior. Children with LOC-E experienced more worries than healthy children even though they could control their behavior best. Corresponding to their lack of control, children with a double diagnosis reported the highest probability to worry about losing control.

Our findings underline the importance of assessing ADHD in children suffering from LOC-E or BED in order to identify those children with a double diagnosis, as a double diagnosis is frequent (above 25% in our sample) and related to multiple self-regulation problems. Future studies including larger sample sizes should specify, whether the effects we found are predominantly related to subtypes of ADHD and should focus more on the role of negative affect prior to the task. If these findings are replicated, it should be tested, whether children with a double diagnosis of LOC-E and ADHD benefit from specific trainings to increase self-regulation of eating behavior.
